# Tailoring Functionalized Lignin-Based Spherical Resins as Recyclable Adsorbents for Heavy Metal Uptake

**DOI:** 10.3390/polym17243324

**Published:** 2025-12-16

**Authors:** Gao Xiao, Shumin Xie, Bizheng Mao, Hong Chen, Yiwei Xue, Qingmei Xu, Jie Guo, Manna Dai

**Affiliations:** 1 College of Environment and Safety Engineering, Fuzhou University, Fuzhou 350108, China; 230620034@fzu.edu.cn (S.X.); 230627080@fzu.edu.cn (B.M.); hongchenfzu@163.com (H.C.); 230627059@fzu.edu.cn (Y.X.); asus08520@163.com (J.G.); 2 School of Advanced Manufacturing, Fuzhou University, Jinjiang 362251, China; 238527054@fzu.edu.cn; 3 Computing & Intelligence Department, Institute of High Performance Computing, Agency for Science, Technology and Research (A*STAR), Singapore 138632, Singapore; manna_dai@ihpc.a-star.edu.sg

**Keywords:** lignin, pulping black liquor, graft copolymerization, sustainable adsorbents, heavy metal removal

## Abstract

A novel mesoporous spherical chelating lignin-based adsorbent was successfully synthesized via inverse suspension polymerization using sulfate pine pulping black liquor as raw material, followed by graft copolymerization with acrylonitrile and subsequent amination. The obtained aminated cyanoethyl spherical lignin resin (ACSLR) exhibited a well-defined porous morphology and abundant active sites, as confirmed by SEM and FT-IR. Adsorption experiments demonstrated high Pb^2+^ uptake capacity (63.98 mg·g^−1^) under optimal conditions (pH = 5.5, 2.0 g·L^−1^ adsorbent dosage, and 150 mg·L^−1^ initial concentration of Pb^2+^ solution). The adsorption process followed the Langmuir isotherm and pseudo-second-order kinetics, indicating monolayer chemisorption dominated by amino and cyano groups. This work provides a sustainable strategy for valorizing industrial lignin waste into efficient adsorbents for heavy metal removal, highlighting its potential for practical wastewater treatment applications.

## 1. Introduction

The pervasive contamination of water resources by heavy metals represents a pressing global environmental challenge, with lead (Pb^2+^) being among the most toxic and persistent pollutants [1,2]. Originating from various industrial activities, Pb^2+^ ions pose severe risks to ecosystems and human health due to their non-biodegradable nature, high bioaccumulation potential, and detrimental effects on neurological, reproductive, and metabolic systems [3]. Conventional methods for removing Pb^2+^ from wastewater, including chemical precipitation, ion exchange, membrane filtration, and electrochemical treatment, often suffer from high operational costs, the generation of secondary waste, or limited efficiency at low metal concentrations [4,5,6]. Among these, adsorption is recognized as a particularly promising approach due to its simplicity, cost-effectiveness, and potential use of renewable resources.

In this context, biosorbents derived from biomass—such as agricultural wastes, forestry byproducts, and polysaccharides—have attracted significant interest as sustainable alternatives to conventional adsorbents [7,8,9]. However, many biomass-based materials, including those derived from bark, chitosan, or rice husk, are typically obtained in powdered or irregular particulate forms [10]. These morphologies often lead to practical limitations, such as poor hydraulic permeability, structural instability in flow systems, and difficulties in separation and regeneration, thereby hindering their large-scale column-based applications [11].

Lignin, the second most abundant natural polymer after cellulose, constitutes a major byproduct of the pulp and paper industry [12]. Traditionally treated as waste, its disposal represents both an economic burden and an environmental concern [13]. Nevertheless, lignin possesses a unique macromolecular structure rich in phenolic hydroxyl, carboxyl, and methoxy groups, offering active sites conducive to chemical modification and metal ion complexation [14]. Functionalization through graft copolymerization, sulfonation, or amination has been employed to enhance its adsorption capacity and selectivity toward heavy metals [15]. Despite these advances, the development of lignin-based materials with uniform morphology, hierarchical porosity, and mechanical robustness remains a considerable materials challenge [16].

Herein, the rational design and synthesis of a novel mesoporous aminated cyanoethyl spherical lignin resin (ACSLR) for efficient Pb^2+^ removal are reported. Using lignin extracted from sulfate pine pulping black liquor—an abundant and undervalued industrial residue—we constructed structurally well-defined spherical beads via inverse suspension polymerization. Epichlorohydrin served as an effective crosslinker, imparting mechanical stability and facilitating the formation of a mesoporous architecture. Subsequent graft copolymerization with acrylonitrile introduced cyano groups, which were further functionalized through amination to yield chelating amino-cyano motifs with high affinity for Pb^2+^ ions. This study systematically investigates the adsorption performance of ACSLR under varying conditions, including pH, adsorbent dosage, initial Pb^2+^ concentration, and contact time. The adsorption mechanisms were elucidated through kinetic and isotherm analyses, complemented by spectroscopic and microscopic evidence. The resulting adsorbent demonstrates not only high capacity and selectivity for Pb^2+^, but also excellent structural integrity suitable for continuous flow operations. Our work underscores the potential of lignin valorization into advanced functional materials, offering a sustainable strategy for “waste-to-wealth” conversion and contributing to the development of green and scalable technologies for water remediation.

## 2. Materials and Methods

### 2.1. General Materials

The pulping black liquor is kraft Masson pine pulping black liquor (with a solid content of 40.1%, provided by Nanping Paper Industry Co., Ltd., Nanping, China). 10# transformer oil, sodium dodecylbenzene sulfonate, epichlorohydrin, acetone, acrylonitrile, hydrogen peroxide, ferrous sulfate, sodium sulfite, potassium persulfate, lead nitrate, ethyl ether, anhydrous methanol, hydroxylamine hydrochloride, anhydrous sodium carbonate, etc. All reagents and solvents were of analytical reagent grade and deionized water was used for preparing the solutions.

### 2.2. Characterizations

Fourier Transform Infrared (FT-IR) spectra of aminated cyanoethyl spherical lignin resin (ACSLR) were obtained using a Thermo Fisher Scientific iS5 spectrometer (Waltham, MA, USA) within 4000–400 cm^−1^ using KBr pellets. The micro-morphology was observed by scanning electron microscope (SEM) measurements were conducted on a Thermo Scientific Apreo 2S field emission (Waltham, MA, USA). The residual Pb^2+^ concentration was quantified using inductively coupled plasma atomic emission spectrometry (ICP-AES; PerkinElmer Optima 2100 DV, Waltham, MA, USA).

### 2.3. Preparation of Porous Spherical Lignin Beads from Pulping Black Liquor

40.0 g of filtered pulping black liquor was charged into a 500 mL three-neck flask equipped with mechanical stirring. To this, three volumes of transformer oil and 0.12 g of sodium dodecylbenzene sulfonate (SDBS) were added as the continuous phase and dispersant, respectively. The mixture was heated to 60 °C under constant stirring. Subsequently, 2.4 g of epichlorohydrin, acting as a crosslinking agent, was introduced dropwise into the system. The reaction was maintained at this temperature for 1 h, then elevated to 90 °C and allowed to proceed for an additional 2 h. After cooling to room temperature, the upper oil phase was recovered for reuse. The reddish-brown product in the lower phase was collected, thoroughly washed with deionized water and acetone, filtered, and dried under ambient conditions to afford porous spherical lignin beads (SLB).

### 2.4. Fabrication of Porous Cyanoethyl-Functionalized Spherical Lignin Beads

**Figure 1:**
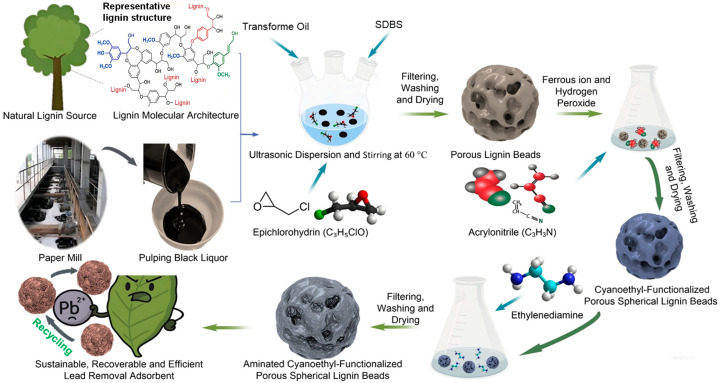
Schematic diagram of the synthesis of porous aminated cyanoethyl-functionalized spherical lignin resin (ACSLR) adsorbent.

10 g of the synthesized porous spherical lignin beads were transferred into a 500 mL conical flask containing 400 mL of distilled water and 7 g of acrylonitrile monomer. The suspension was agitated at 350 rpm and 55 °C for 30 min to ensure homogeneous dispersion. Graft copolymerization was initiated by introducing 0.9 mmol of Fe^2+^ and a stoichiometric amount of H_2_O_2_. The reaction was continued for 90 min under the same conditions. The resulting product was isolated via filtration, washed sequentially with water, acetone, and diethyl ether, and air-dried to yield cyanoethyl-modified spherical lignin beads (denoted as CSLB). A schematic illustration of the step-wise synthesis is provided in Figure 1.

### 2.5. Preparation of Aminated Cyanoethyl-Functionalized Spherical Lignin Resin

5 g of the cyanoethyl-modified lignin beads were immersed in a mixture of 50 mL distilled water and 10 g ethylenediamine in a 500 mL conical flask. The system was shaken for 10 min at room temperature in a constant-temperature incubator. Pre-determined quantities of sodium hydroxide and formaldehyde solutions were then introduced sequentially. The amination reaction was carried out at 60 °C for 3 h under high-shear mixing. The final product was collected by filtration, washed extensively with water, ethanol, and again with water, and dried at 60 °C to obtain the aminated cyanoethyl-functionalized spherical lignin resin (denoted as ACSLR, Figure 1).

### 2.6. Adsorption Experiments of Pb^2+^ Using ACSLR Adsorbent

The batch adsorption experiments were conducted by introducing a predetermined mass of the aminated cyanoethyl-functionalized spherical lignin resin (ACSLR) into 100 mL of an aqueous Pb^2+^ solution of varying initial concentrations (50–250 mg·L^−1^) contained in a 250 mL sealed conical flask. The suspensions were adjusted to optimal pH 5.5 using 0.1 mol·L^−1^ NaOH or HNO_3_ solutions as required, then agitated at 350 rpm in a temperature-controlled shaker at 25 °C for 4 h to ensure equilibrium attainment. Parallel kinetic studies performed at 150 mg·L^−1^ initial concentration with sampling intervals from 5 min to 4 h enabled adsorption rate determination. All experiments included duplicate controls and blank solutions. Take a proper amount of Pb(II) simulated wastewater treated by ACSLR adsorbent, dilute it 10 times with 5% nitric acid solution after centrifugation, and manually inject it with an inductively coupled plasma atomic emission spectrometer (ICP-AES) to determine the mass concentration of residual lead ions in each water sample.

The adsorption capacity (*q_e_*) of ions was calculated according to Equation (1) [17]:

(1) $${\;\;\;\;\;\;\;\;\;\;\;\;\;\;q}_{e}=\frac{\left(C_{0}-C_{t}\right)V}{m}$$

where *q_e_* represent the equilibrium adsorption capacity (mg·g^−1^), *C*_0_ is the initial concentration of Pb^2+^ in the solution (mg·L^−1^), *C_t_* is the concentration of Pb^2+^ at adsorption equilibrium (mg·L^−1^), *V* is the volume of the initial solution (mL), *m* is the mass of the adsorbent material (mg).

### 2.7. Adsorption Kinetics Studies of Pb^2+^ Removal by ACSLR Adsorbent

Adsorption kinetics were evaluated by monitoring the uptake of Pb^2+^ at various time intervals using a fixed adsorbent dosage of 2 g·L^−1^ ACSLR in 100 mL of Pb(II) solution (150 mg·L^−1^). The residual metal ion concentration was measured at predetermined time points. For adsorption kinetics analysis, batch experiments were performed at different temperatures. Samples (2 mL) were withdrawn at specific time intervals, immediately filtered through 0.45 μm membranes, and analyzed for residual Pb^2+^ concentration using atomic absorption spectroscopy. The kinetic data were and analyzed fitted to the Pseudo-First-Order (PFO) Model (This model assumes adsorption rate is proportional to the number of unoccupied sites, following a first-order dependence on the difference between equilibrium and transient adsorption capacities.) and Pseudo-Second-Order (PSO) Models (This model presumes the adsorption rate is controlled by chemisorption mechanisms, with rate dependence on the square of the number of available active sites.) using linear regression analysis [18].

### 2.8. Adsorption Isotherm Analysis of Pb^2+^ Removal by ACSLR Adsorbent

Adsorption isotherm experiments were conducted to evaluate the equilibrium characteristics of Pb(II) uptake by ACSLR under varying temperatures. Specifically, 2 g·L^−1^ of the adsorbent was introduced into simulated wastewater samples with different initial concentrations of Pb(II), prepared in accordance with previously optimized adsorption conditions. The systems were equilibrated at 30, 40, and 50 °C (303, 313, and 323 K), respectively. After reaching adsorption equilibrium, the residual Pb(II) concentrations were determined, and the corresponding equilibrium adsorption capacities were calculated. The equilibrium adsorption data were fitted to the Langmuir and the Freundlich isotherm models using linear regression analysis [19]. The Langmuir model, which assumes monolayer adsorption on a homogeneous surface, is expressed as [20]:

(2) $$\frac{C_{e}}{q_{e}}=\frac{C_{e}}{q_{max}}+\frac{1}{q_{max}K_{L}}$$

where *q_e_* is the equilibrium adsorption capacity (mg/g), *C_e_* is the equilibrium concentration of Pb(II) (mg·L^−1^), *q_max_* represents the maximum adsorption capacity (mg·g^−1^), and *K_L_* is the Langmuir equilibrium constant (L/mg).

The Freundlich model, which describes multilayer adsorption on heterogeneous surfaces [21], is given by:

(3) $$\ln q_{e}=\ln K_{f}+\frac{\ln C_{e}}{n}$$

where *K_f_* (L·mg^−1^) is the Freundlich constant related to adsorption capacity, and *n* (dimensionless) is an empirical constant indicating adsorption intensity.

### 2.9. Desorption and Regeneration Studies of ACSLR Adsorbent for Pb^2+^ Removal

The Pb^2+^-laden material was immersed in 50 mL 0.1 M HNO_3_ desorbing solution and agitated at 350 rpm for 24 h at 25 °C. After desorption, the regenerated adsorbent was thoroughly rinsed with deionized water until a neutral pH was achieved, then dried at 60 °C for subsequent reuse cycles. Desorption efficiency was calculated based on Pb^2+^ concentration in the eluent measured by ICP-AES. Four consecutive adsorption–desorption cycles were performed with 0.1 M HNO_3_ as the optimal eluent to evaluate long-term reusability and capacity retention.

## 3. Results and Discussion

As schematically illustrated in Figure 1, a novel lignin-based adsorbent was successfully synthesized through a sustainable and innovative route using sulfate pine pulping black liquor—an abundant industrial waste—as the raw material. Initially, spherical lignin beads (SLB) with uniform morphology were fabricated via inverse suspension polymerization, providing a robust macroporous scaffold for further functionalization. Subsequently, graft copolymerization with acrylonitrile was efficiently initiated by a Fe^2+^/H_2_O_2_ redox system under high-shear conditions, leading to the formation of cyanoethyl-modified spherical lignin (CSLB). This step introduced cyano groups, which serve as key precursors for further chemical modification. Finally, through a Mannich-type reaction, amination of the cyanoethyl lignin yielded the target product—aminated cyanoethyl spherical lignin resin (ACSLR) adsorbent —equipped with abundant amino and cyano functional groups conducive to metal ion coordination. The structural evolution during each modification step was corroborated by spectroscopic and microscopic analyses (e.g., FT-IR, SEM), confirming the successful incorporation of functional groups and preservation of the spherical macroporous architecture. The resulting ACSLR adsorbent exhibits promising potential as an effective adsorbent for heavy metal removal, particularly for Pb(II), owing to its high surface functionality, sustainable origin, and engineered morphology conducive to rapid ion uptake and facile separation [22]. This combination of waste valorization and precise chemical tailoring underscores the dual environmental and functional appeal of this material, highlighting a strategic advance in the design of high-performance biosorbents.

### 3.1. Morphology Analysis of ACSLR Before and After Pb^2+^ Adsorption

**Figure 2:**
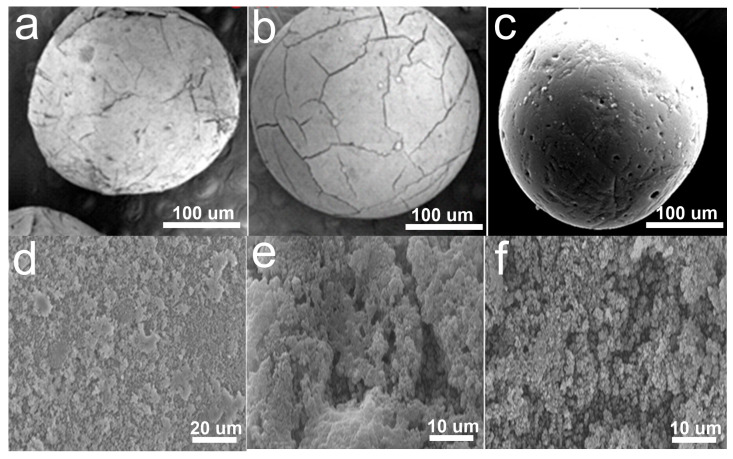
(**a**) SEM image of SLB; (**b**) SEM image of CSLB; (**c**) SEM image of ACSLR; (**d**) SEM image of the cross-sectional surface topography of SLB; (**e**) SEM image of the cross-sectional surface topography of ACSLR; (**f**) SEM image of the cross-sectional surface topography of ACSLR after adsorbing Pb^2+^.

Figure 2 presents scanning electron microscopy (SEM) images illustrating the morphological evolution of the aminated cyanoethyl spherical lignin resin (ACSLR) adsorbent before and after Pb^2+^ adsorption. As depicted in Figure 2a, the pristine spherical lignin beads (SLB), synthesized from sulfate softwood pulping black liquor via reverse-phase suspension polymerization, exhibit a well-defined spherical architecture with a macroporous surface structure. Following graft copolymerization with acrylonitrile, the resulting cyanoethyl-functionalized lignin beads (CSLB) retain their spherical integrity but display a notably rougher surface topology and increased surface porosity (Figure 2b). Subsequent amination further modifies the morphology, yielding ACSLR with a denser, more consolidated surface and a homogeneously distributed porous network (Figure 2c). Cross-sectional analysis reveals that the internal structure of the unmodified SLB is loose and uniformly porous (Figure 2d), whereas the grafted and aminated CSLB exhibits a more complex, interwoven morphology with reduced smoothness, suggesting the successful incorporation and aggregation of polyacrylonitrile chains (Figure 2e). After exposure to Pb^2+^, the surface of ACSLR becomes significantly rougher, with pores appearing densely occupied by particulate deposits (Figure 2f). This marked morphological alteration is attributed to the introduction of abundant active functional groups (e.g., amino and cyano moieties) during chemical modification, which not only increase the molecular weight and crosslinking density but also provide numerous chelating sites for Pb^2+^ uptake [23]. The visible deposition of lead-containing species within the pores confirms effective metal ion adsorption and correlates with the enhanced adsorption capacity of the functionalized resin.

### 3.2. The Molecular Level Confirmation of the Adsorbent Synthesized by Step-by-Step Modification

**Figure 3:**
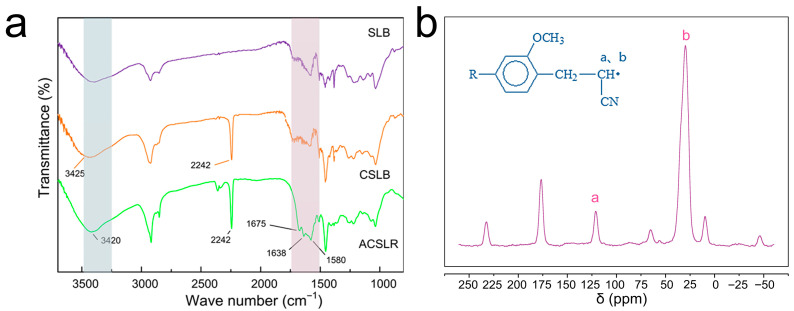
The successful modification and synthesis of ACSLR adsorbent were confirmed at the molecular structure level. (**a**) Comparative FT-IR spectra of SLB, CSLB and ACSLR (The colored background box highlights the change of wave number in this area more clearly); (**b**) The ^13^C-NMR graph of ACSLR (peak a and peak b respectively corresponding to the carbon atoms of the cyano group (–C≡N) and the methylene group).

Figure 3a displays the comparative FTIR spectra of SLB, CSLB, and ACSLR. All three samples exhibit a distinct absorption peak near 3420 cm^−1^, corresponding to O–H stretching vibrations, indicating the presence of abundant hydroxyl groups in the lignin-based spherical adsorbent (LSA) cross-linked with epichlorohydrin. As shown in Figure 3a, most of the original functional groups of SLB are largely retained in both CSLB and ACSLR. CSLB shows a characteristic –C≡N stretching vibration peak at 2242 cm^−1^, while no absorption band associated with carbon–carbon double bonds is observed around 1650 cm^−1^, confirming the successful grafting of cyano groups onto SLB to form CSLB. Notable spectral differences appear in the 1600–1700 cm^−1^ region for ACSLR compared to SLB and CSLB. Specifically, ACSLR exhibits –NH stretching vibration peaks at 1580 cm^−1^, 1638 cm^−1^, and 1675 cm^−1^. Furthermore, compared to CSLB, the broadened absorption band in the 3250–3600 cm^−1^ range, together with the emergence of a –NH bending vibration near 1640 cm^−1^, indicates that the absorption in the 3250–3600 cm^−1^ region is attributed not only to O–H stretching but also to –NH stretching vibrations. These results confirm the successful introduction of amine functional groups, yielding ACSLR. On the other hand, the ^13^C NMR spectrum of ACSLR is presented in Figure 3b. Two distinct resonance signals are observed at δ = 117.7 ppm (peak a) and 20.7 ppm (peak b), corresponding to the carbon atoms of the cyano group (–C≡N) and the methylene group adjacent to it, respectively. These signals confirm the successful introduction of cyanoethyl groups into the spherical lignin matrix. Additionally, a characteristic signal at δ = 161.3 ppm is attributed to the C–O carbon within the lignin backbone. The remaining resonances in the spectrum are consistent with aromatic carbons and associated C–H environments, all characteristic of the fundamental lignin framework. These collective findings provide conclusive evidence for the successful synthesis of the ACSLR adsorbent.

### 3.3. The Characterization of the Mesoporous Structure of ACSLR Adsorbent

**Figure 4:**
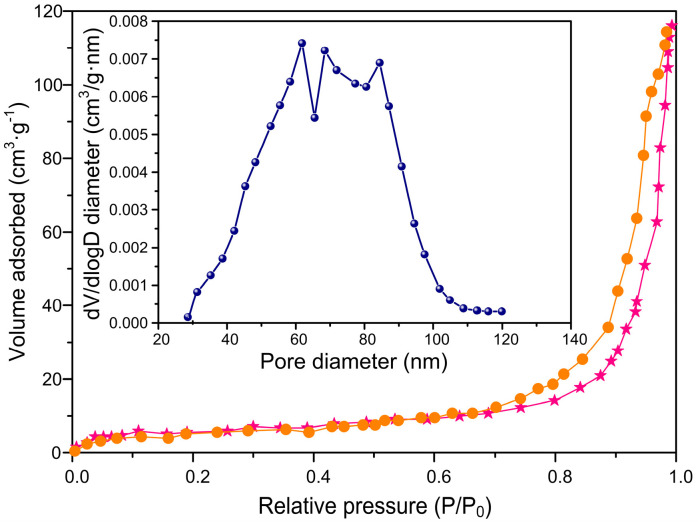
N_2_ adsorption–desorption isotherms with corresponding Barrett–Joyner–Halenda (BJH) pore size distribution plots (Inset illustration shows the pore size distribution of the ACSLR sample), demonstrating mesoporous characteristics of the ACSLR sample (The hysteresis loop observed between the yellow adsorption branches and pink desorption branches of a nitrogen physisorption isotherm).

The spherical lignin-based adsorbent exhibits a well-developed mesoporous architecture, as confirmed by N_2_ adsorption–desorption isotherm analysis (Shown in Figure 4). The material demonstrates a type-IV isotherm with a distinct H1-type hysteresis loop, indicative of a highly uniform mesoporous structure with narrow pore size distribution (50–90 nm). This optimized mesoporous framework provides abundant accessible binding sites and facilitates efficient mass transfer during adsorption processes. The integration of a well-defined mesoporous structure with the inherent functionality of lignin creates a highly effective adsorbent for heavy metal remediation. The tailored pore system at 50–90 nm proves crucial for achieving both high capacity and fast kinetics for Pb^2+^ uptake, positioning this material as a promising candidate for wastewater treatment applications.

### 3.4. Comparison of Infrared Analysis of ACSLR Before and After Pb^2+^ Adsorption

**Figure 5:**
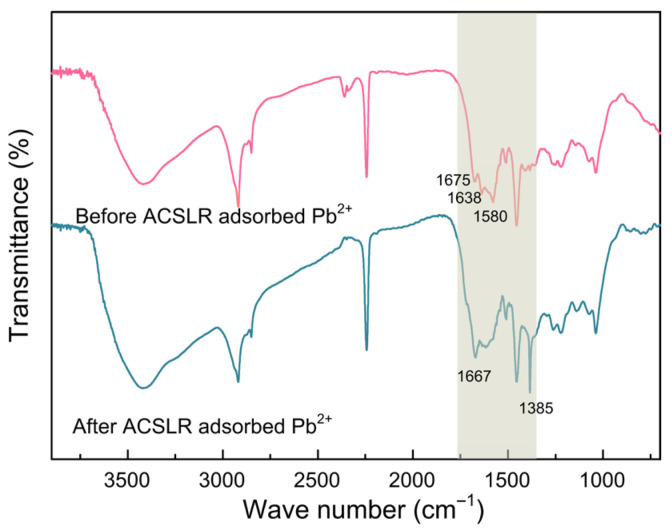
Comparative FT-IR spectra of porous aminated cyanoethyl spherical lignin adsorption resin before and after Pb^2+^ adsorption (The colored background box highlights the change of wave number in this area more clearly).

FT-IR spectroscopy was employed to elucidate the adsorption mechanism of Pb(II) onto the functionalized ACSLR microspheres. A comparison of ACSLR before and after Pb(II) adsorption is shown in Figure 5. The spectrum of pristine ACSLR exhibits a broad absorption band at approximately 3425 cm^−1^, attributable to O–H stretching vibrations [24]. This feature originates from the abundant hydroxyl groups introduced through cross-linking with epichlorohydrin during resin synthesis. Notable spectral changes are observed in the range of 1500–1700 cm^−1^ after Pb(II) adsorption, which are associated with N–H stretching vibrations [25]. The original peaks at 1675, 1638, and 1580 cm^−1^ characteristic of free or weakly hydrogen-bonded –NH groups—either disappear significantly or shift upon metal uptake. Specifically, a new peak emerges at 1667 cm^−1^, indicating chemical coordination between nitrogen-containing ligands and Pb(II) ions. These changes suggest that –NH groups play a decisive role in the chelation process, likely through the formation of stable complexes between amine functionalities and divalent lead ions. Furthermore, the emergence of a new absorption band at ~1385 cm^−1^ can be attributed to the formation of Pb-O bonds. The observed spectral shifts and intensity reductions provide strong evidence that chemisorption, rather than physisorption, dominates the adsorption process. The grafted amine groups on ACSLR exhibit high specificity toward Pb(II), underscoring the effectiveness of chemical modification in enhancing both the selectivity and capacity of the lignin-based adsorbent [26].

### 3.5. The Adsorption Law of Lead Ion Removal by ACSLR

**Figure 6:**
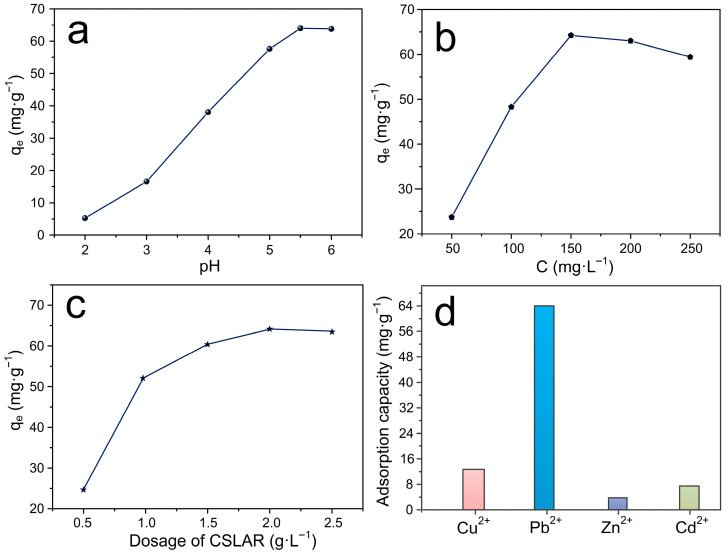
(**a**) Effect of pH value on Pb^2+^ adsorption capacity of ACSLR adsorbent; (**b**) Effect of initial lead ion concentration on Pb^2+^ adsorption by ACSLR; (**c**) Effect of adsorbent dosage on the adsorption performance of Pb^2+^ by ACSLR; (**d**) The Competitive adsorption capacities of Cu^2+^, Pb^2+^, Zn^2+^ and Cd^2+^ in their quaternary metal solution (Initial concentration of M^2+^: 1.0 mmol·L^−1^; ACSLR dosage: 2.0 g·L^−1^; contact time: 4 h, temperature: 25 °C).

The solution pH exerts a critical influence on the adsorption behavior of heavy metal ions due to its impact on both the ionization state of functional groups and the metal speciation. As illustrated in Figure 6a, the adsorption capacity of ACSLR adsorbent for Pb^2+^ was evaluated over a pH range of 2.0–6.0 under fixed conditions: initial Pb^2+^ concentration = 150 mg·L^−1^, adsorbent dosage = 2.0 g·L^−1^, temperature = 30 °C, and contact time = 4 h. The adsorption capacity increased markedly from 5.23 mg/g at pH 2.0 to 63.98 mg·g^−1^ at pH 5.5. This pronounced pH dependence can be attributed to the protonation of surface functional groups (–OH, –NH_2_) under highly acidic conditions, which renders the adsorbent surface positively charged and thus electrostatically unfavorable for Pb^2+^ uptake. As the pH increases, deprotonation enhances the availability of binding sites, enabling more effective coordination through lone electron pairs on nitrogen and oxygen atoms. Specifically, chelation via amine groups and Schiff base motifs likely contributes to the high affinity between ACSLR adsorbent and Pb^2+^. Beyond pH 6.0, precipitation of Pb(II) as hydroxide species (e.g., Pb(OH)_2_ or [Pb(OH)_3_]^−^) becomes significant, complicating the interpretation of adsorption measurements. Therefore, pH 5.5 was identified as the optimum value for subsequent experiments, ensuring maximal adsorption efficiency while avoiding precipitation-related artifacts. These results underscore the crucial role of surface charge and ligand accessibility in the metal ion uptake process and highlight the effectiveness of ACSLR adsorbent’s functionalized structure under mildly acidic conditions [27].

The influence of initial Pb^2+^ concentration on the adsorption performance of ACSLR adsorbent was systematically investigated under optimized conditions (pH = 5.5, adsorbent dosage = 2.0 g·L^−1^, T = 30 °C, t = 4 h). As depicted in Figure 6b, the adsorption capacity of ACSLR adsorbent increased progressively with rising initial Pb^2+^ concentration up to 150 mg·L^−1^, reaching a maximum equilibrium uptake of 63.98 mg·g^−1^. Beyond this threshold, further increases in concentration did not lead to significant enhancement in adsorption capacity, indicating the onset of saturation behavior. This concentration-dependent uptake profile is characteristic of adsorbents with a finite number of active sites. At lower concentrations, the abundant availability of binding sites relative to Pb^2+^ ions results in high adsorption efficiency. As the concentration increases, the driving force for mass transfer is enhanced, leading to greater uptake until the active sites are fully utilized. However, at elevated concentrations (>150 mg·L^−1^), several factors may contribute to the observed plateau: (1) increased cationic competition and charge shielding effects reduce the effective affinity between Pb^2+^ and negatively charged functional groups on lignin; (2) potential agglomeration of ACSLR adsorbent under high ion strength may cause conformational changes, such as curling of polymer chains, leading to the encapsulation of active groups (e.g., phenolic hydroxyl and carboxyl groups); and (3) limited accessibility to adsorption sites due to surface overcrowding and pore blockage. These results underscore the importance of initial concentration in optimizing adsorption performance and highlight the balanced interplay between mass transfer driving forces and site availability in the efficient removal of Pb^2+^ using functionalized lignin-based adsorbents [28].

The influence of ACSLR adsorbent dosage on the adsorption performance was evaluated using 100 mL of a 150 mg/L Pb^2+^ solution at pH 5.5 and 30 °C with a contact time of 4 h. As summarized in Figure 6c, both the removal efficiency and equilibrium adsorption capacity exhibited strong dependence on adsorbent loading. When the ACSLR adsorbent dosage was increased from 0.5 to 2.5 g·L^−1^, the Pb^2+^ removal efficiency rose significantly, accompanied by an increase in the equilibrium adsorption capacity from 24.63 to 63.98 mg·g^−1^. This trend can be attributed to the greater availability of active binding sites at higher adsorbent loads, which enhances metal ion accessibility and facilitates uptake [29]. However, beyond the dosage of 2.0 g·L^−1^, the adsorption capacity reached a plateau, and further increases in ACSLR adsorbent amount did not yield substantial improvements in Pb^2+^ removal. This saturation behavior suggests that at higher adsorbent concentrations, overlapping or aggregation of resin particles may reduce the effective surface area and active site utilization—a phenomenon often associated with particle crowding and site masking. Moreover, the constant residual Pb^2+^ concentration beyond the optimal dosage reflects the achievement of adsorption–desorption equilibrium under given conditions. These findings highlight the importance of optimizing adsorbent dosage to maximize efficiency while minimizing material usage. The selection of 2.0 g·L^−1^ ACSLR adsorbent represents a balance between high removal performance and economic feasibility, rendering it suitable for practical applications in heavy metal remediation.

To evaluate the practical applicability of ACSLR, its adsorption selectivity was investigated in a multi-component system containing competing divalent cations (Pb^2+^, Cd^2+^, Cu^2+^, and Zn^2+^). As illustrated in Figure 6d, ACSLR exhibited remarkable selectivity toward Pb^2+^, demonstrating a significantly higher adsorption capacity and selectivity coefficient compared to other metal ions. The exceptional selectivity for Pb^2+^ can be attributed to its distinct physicochemical properties among the tested divalent cations. Pb^2+^ possesses relatively high electronegativity and low hydration enthalpy, which enhances its intrinsic affinity for oxygen- and nitrogen-containing functional groups present in the adsorbent matrix. These characteristics facilitate stronger coordinate bonds and more favorable ion-exchange interactions with the active sites of ACSLR. The results clearly indicate that the adsorption preference follows the order: Pb^2+^ >> Cu^2+^ > Cd^2+^ > Zn^2+^, confirming that the adsorbent maintains its targeting capability even in complex multi-metal environments. This pronounced selectivity, coupled with the high uptake capacity, underscores the potential of ACSLR for precise and efficient removal of lead ions from real wastewater systems containing multiple competing contaminants.

### 3.6. Adsorption Kinetics Analysis

**Figure 7:**
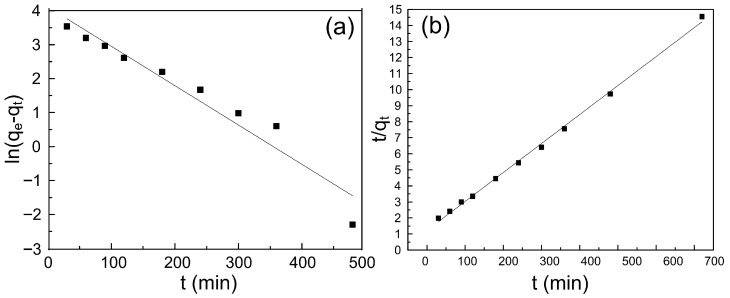
(**a**) Pseudo-first-order kinetic fitting curve of removing Pb^2+^ by ACSLR adsorbent; (**b**) Pseudo-second-order kinetic equation fitting curve of removing Pb^2+^ by ACSLR adsorbent.

The adsorption kinetics of Pb(II) onto ACSLR adsorbent were investigated using pseudo-first-order and pseudo-second-order models, with the corresponding fitting results illustrated in Figure 7a,b. Linear regression analysis clearly indicates that the adsorption process is better described by the pseudo-second-order model, which exhibits a significantly higher correlation coefficient (R^2^ = 0.998) compared to that of the pseudo-first-order model (R^2^ = 0.939). Furthermore, the theoretical equilibrium adsorption capacity (*q_e_*, cal) derived from the pseudo-second-order model (52.3 mg∙g^−1^) aligns closely with the experimentally determined value (50 mg∙g^−1^), whereas the pseudo-first-order model overestimates the capacity (61.1 mg∙g^−1^) (shown in Table S1). The superior agreement of the pseudo-second-order model with experimental data suggests that the adsorption process is predominantly governed by chemisorption, involving valence forces through sharing or exchange of electrons between Pb(II) ions and functional groups on ACSLR adsorbent, such as amine and cyano groups. This kinetic behavior underscores the effectiveness of the chemically modified lignin surface in facilitating rapid and efficient metal ion uptake [30,31].

### 3.7. Adsorption Isotherm Analysis

**Figure 8:**
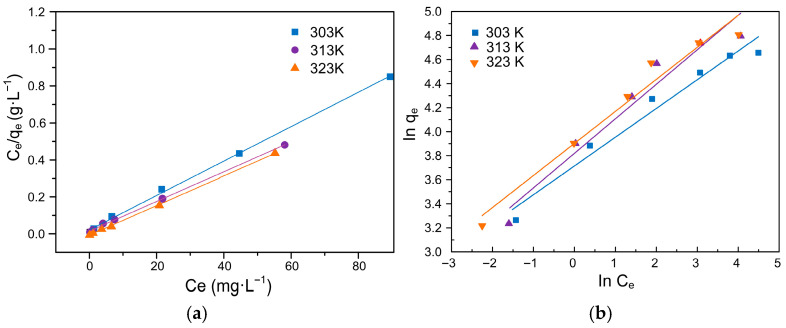
(**a**) Fitting graph of the Langmuir Equation; (**b**) Fitting graph of the Freundlich equation.

Nonlinear regression was employed to determine the best-fitting model and corresponding parameters, providing insight into the adsorption mechanism and affinity between Pb(II) and the functionalized lignin adsorbent [32]. As shown in Figure 8, Tables S2 and S3, analysis of the adsorption isotherms using the Langmuir and Freundlich models revealed that the Langmuir model regression coefficient (R_L_^2^ ≥ 0.998) was higher than that of the Freundlich model (R_F_^2^ ≥ 0.945), indicating that the Langmuir model provides a better fit to the experimental data. Based on these results, it can be concluded that the adsorption of Pb^2+^ by ACSLR adsorbent follows the Langmuir model, suggesting that the process occurs primarily via monolayer adsorption dominated by chemisorption. Furthermore, the increase in the Langmuir constant b with rising temperature indicates enhanced binding affinity between Pb^2+^ and ACSLR adsorbent [32].

### 3.8. Thermodynamic Study on the Adsorption of Pb(II) onto ACSLR

The adsorption thermodynamics of Pb(II) onto ACSLR were investigated by determining the Gibbs free energy change (ΔG), enthalpy change (ΔH), and entropy change (ΔS) (Shown in Figure S1). The thermodynamic parameters summarized in Table S4 were derived from the temperature-dependent adsorption isotherms measured between 303 K and 323 K. The negative values of ΔG observed at all temperatures confirm the spontaneous nature of the adsorption process. The magnitude of ΔG becomes more negative with increasing temperature, indicating enhanced adsorption spontaneity at elevated temperatures. The positive ΔH value (+14.63 kJ·mol^−1^) reveals the endothermic character of Pb(II) uptake, which is further confirmed by the increased adsorption capacity with rising temperature. The positive ΔS value (+142.79 J·mol^−1^·K^−1^) suggests an increase in randomness at the solid–liquid interface during adsorption, reflecting the displacement of solvated water molecules by metal ions and the consequent release of structured water into the bulk solution. The combination of positive ΔH and positive ΔS values indicates that the adsorption process is driven by both enthalpy and entropy contributions, with the entropy term playing a particularly significant role in governing spontaneity. These thermodynamic characteristics, along with the measured ΔH magnitude, suggest that Pb(II) adsorption onto ACSLR proceeds primarily through chemisorption mechanisms.

### 3.9. The Reusability and Stability of ACSLR Adsorbent for Removing Pb^2+^

**Figure 9:**
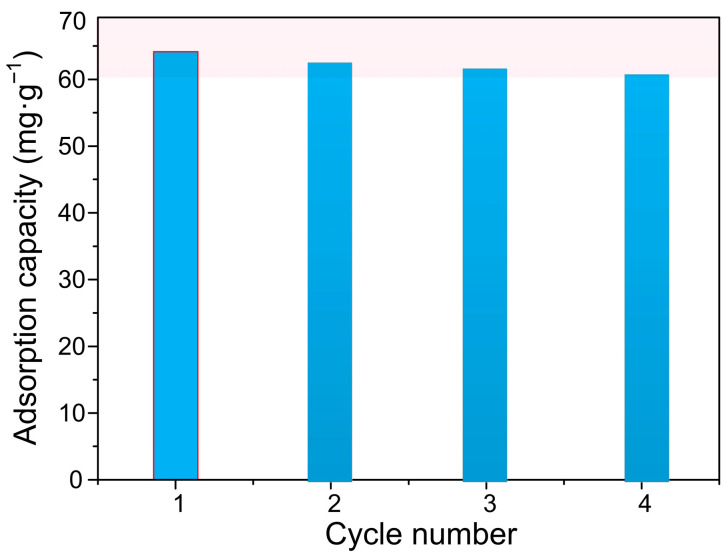
The reusability of ACSLR adsorbent for removing Pb^2+^ (Initial concentration of Pb^2+^: 150 mg·L^−1^; ACSLR dosage: 2.0 g·L^−1^; pH: 5.5).

The reusability of the aminated cyanoethyl spherical lignin resin (ACSLR) was systematically evaluated over multiple adsorption–desorption cycles to assess its practical applicability. As depicted in Figure 9, the adsorbent exhibited higher Pb^2+^ adsorption capacity compared with other common adsorbents (Table S5) and remarkable stability, with only a marginal decline in Pb^2+^ removal efficiency after four consecutive regeneration cycles. The adsorption capacity retained approximately 95.3% of its original value, corresponding to a minimal loss of 4.7%. This outstanding recyclability can be attributed to the robust chemical and mechanical stability imparted by the cross-linked lignin framework and the covalently grafted functional groups, which remain largely intact throughout the regeneration process. The slight reduction in performance may be associated with minor losses of active sites or partial pore blockage after repeated use [33]. These results underscore the potential of ACSLR as a durable and economically viable adsorbent for long-term application in heavy metal removal from wastewater.

## 4. Conclusions

In summary, a novel aminated cyanoethyl spherical lignin resin (ACSLR) was successfully fabricated from industrial pulping black liquor via inverse suspension polymerization, grafting, and amination. The adsorbent exhibits excellent Pb^2+^ adsorption capacity (63.98 mg·g^−1^) under optimized conditions (pH = 5.5, dosage = 2.0 g·L^−1^, C_0_ = 150 mg·L^−1^). Adsorption kinetics and isotherms conform to the pseudo-second-order and the Freundlich models, respectively, indicating a chemisorption-dominated multilayer process. The presence of amino and cyano groups significantly enhances metal ion coordination and selectivity. This work not only offers a high-performance, sustainable adsorbent for heavy metal remediation but also demonstrates a viable pathway for valorizing lignin waste into advanced functional materials, contributing to green wastewater treatment and circular economy strategies.

## Data Availability

The original contributions presented in this study are included in the article/Supplementary Materials. Further inquiries can be directed to the corresponding authors.

## Supplementary Materials

The following supporting information can be downloaded at: https://www.mdpi.com/article/10.3390/polym17243324/s1, References [34,35,36,37] are cited in the supplementary materials. Figure S1. The thermodynamic curve fitting. Table S1. Fitting parameters of Pb^2+^ adsorption kinetics of ACSLR adsorbent; Table S2. Langmuir adsorption isothermal formulas at different temperatures; Table S3. Freundlich adsorption isothermal formulas at different temperatures. Table S4. The thermodynamic parameters. Table S5 Comparison of adsorption parameter of different adsorbent materials for Pb^2+^ removal.
