# Supplementary material for: Tailoring Functionalized Lignin-Based Spherical Resins as Recyclable Adsorbents for Heavy Metal Uptake
Source: Polymers (Basel). 2025 Dec 16;17(24):3324. doi: 10.3390/polym17243324 (PMC12737322; doi:10.3390/polym17243324)
Supplement: Supplementary file 1 [file polymers-17-03324-s001.zip › polymers-3876796-supplementary.pdf]

Supplementary Information

Tailoring Functionalized Lignin-Based Spherical Resins as Recyclable Adsorbents for Heavy Metal Uptake

Gao Xiao[1,2,*], Shumin Xie[1], Bizheng Mao[1], Hong Chen[1], Yiwei Xue[1], Qingmei Xu[2], Jie Guo[1], Manna Dai[3]

[1]*College of Environment and Safety Engineering, Fuzhou University, Fuzhou 350108, Fujian, P. R. China*

[2]*School of Advanced Manufacturing, Fuzhou University, Jinjiang 362251, Fujian, P. R. China*

[3]*Computing & Intelligence Department, Institute of High Performance Computing, Agency for Science, Technology and Research (A*STAR), 138632, Singapore*

*Correspondence to: Prof. Dr. xiaogao@fzu.edu.cn

Section 1. Supplementary Figures and Tables

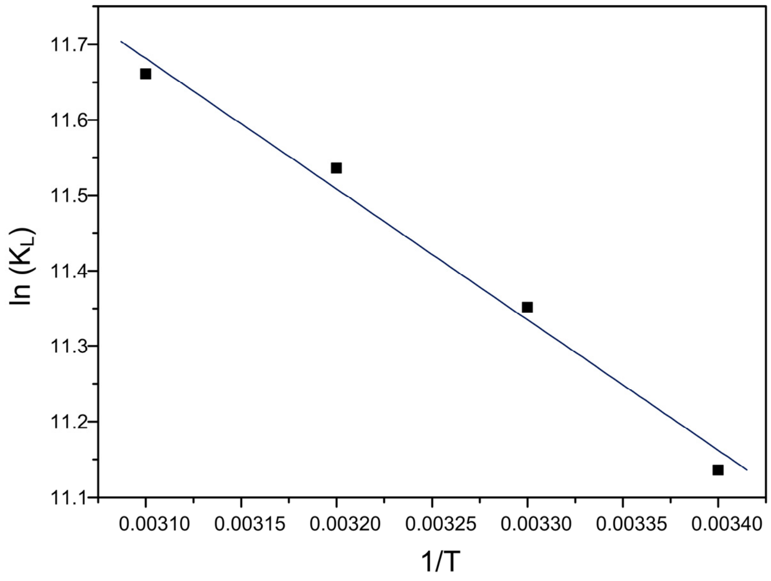

Figure S1 The thermodynamic curve fitting

Table S1. Fitting parameters of Pb^{2+} adsorption kinetics of ACSLR adsorbent.

Adsorbate	Fit equation	K_1/ min^{-1}	K_2/g·(mg ·min)$^{-1}$	Q_e/ mg·g^{-1}	R^2
Pb(Ⅱ)	$ln(Q_e-Q_t) = 4.1109 - 0.0116t$	0.027	---	61.08	0.939
	$t/Q_t = 0.0191t + 1.2231$	---	0.0002	52.29	0.998

Table S2. Langmuir adsorption isothermal formulas at different temperatures.

Adsorbate	Temp	Qm/(mg/g)	K_L/(L/mg)	$C_e/Q_e = 1/(K_L \cdot Q_m) + C_e/Q_m$	R_L^2
	303 K	107.4	0.426	$C_e/Q_e = 0.0218 + 0.0093C_e$	0.998
Pb(Ⅱ)	313 K	125.1	0.538	$C_e/Q_e = 0.0149 + 0.0080C_e$	0.999
	323 K	125.1	0.605	$C_e/Q_e = 0.0132 + 0.0080C_e$	0.999

Table S3. Freundlich adsorption isothermal formulas at different temperatures.

Adsorbate	Temp	K_F	1/n	$lnQ_e = lnK_F + 1/n\ lnC_e$	R^2
	303 K	41.1	0.239	$lnQ_e = 3.7145 + 0.2393lnC_e$	0.957
Pb(Ⅱ)	313 K	45.5	0.287	$lnQ_e = 3.8197 + 0.2873lnC_e$	0.945
	323 K	49.6	0.267	$lnQ_e = 3.9020 + 0.2666lnC_e$	0.965

Table S4. The thermodynamic parameters

Adsorbate	Temp/K	ΔG/KJ·mol-1	ΔS/J·(K·mol)-1	ΔH/KJ·mol-1
Pb(Ⅱ)	293 K	-27.18	141.64	14.36
	303 K	-28.57		
	313 K	-29.94		
	323 K	-31.36		

Table S5 Comparison of adsorption parameter of different adsorbent materials for Pb^{2+} removal

Adsorbent name	pH,	Equilibrium time	Adsorbent dose	Adsorption capacity	Ref
Aminated Cyanoethyl Spherical Lignin Resin (ACSLR)	5.5	60 min	2 g/L	63.98 $mg{\cdot}g^{-1}$	This Work
Nano-silversol-coated Activated Carbon	5.5	60 min	35 g/L	23.81 $mg{\cdot}g^{-1}$	34
Amino-functional Large-size Mesoporous Silica Spheres	4.0	60 min	1 g/L	48.7 $mg{\cdot}g^{-1}$	35
Activated Sludge	6.0	30 min	1 g/L	18.35 $mg{\cdot}g^{-1}$	36
Amino Group's Functionalized Montmorillonite	5.5	90 min	4 g/L	61.1 $mg{\cdot}g^{-1}$	37

[34] Kumar, P. S.; Vincent, C.; Kirthika, K.; Kumar, K. S. Kinetics and equilibrium studies of Pb^{2+} ion removal from aqueous solutions by use of nano-silversol-coated activated carbon. Brazilian Journal of Chemical Engineering 2010, 27(2): 339-346.

[35] Li, P.; Wang, J.; Li, X.; Zhu, W.; He, S.; Han, C.; Luo, Y.; Ma, W.; Liu, N.; Dionysiou, D. D. Facile synthesis of amino-functional large-size mesoporous silica sphere and its application for Pb^{2+} removal. Journal of Hazardous Materials 2019, 378, 120664.

[36] Liu, G.; Tang, X.; Yuan, J.; Li, Q.; Qi, L.; Wang, H.; Ye, Z.; Zhao, Q. Activated sludge process enabling highly efficient removal of heavy metal in wastewater. Environmental Science and Pollution Research 2023, 30(8): 21132-21143

[37] Wang, A.; Chu, Y.; Muhmood, T.; Xia, M.; Xu, Y.; Yang, L.; Lei, W.; Wang, F. Adsorption properties of Pb^{2+} by amino group's functionalized montmorillonite from aqueous solutions. Journal of Chemical & Engineering Data 2018, *63*(8): 2940-2949.
